# Development and validation of a dementia risk score in the UK Biobank and Whitehall II cohorts

**DOI:** 10.1136/bmjment-2023-300719

**Published:** 2023-08-21

**Authors:** Melis Anatürk, Raihaan Patel, Klaus P. Ebmeier, Georgios Georgiopoulos, Danielle Newby, Anya Topiwala, Ann-Marie G de Lange, James H Cole, Michelle G Jansen, Archana Singh-Manoux, Mika Kivimäki, Sana Suri

**Affiliations:** 1 Centre for Medical Image Computing, Department of Computer Science, University College London, London, UK; 2 Department of Psychiatry, University of Oxford, Oxford, UK; 3 Oxford Centre for Human Brain Activity, Wellcome Centre for Integrative Neuroimaging, University of Oxford, Oxford, UK; 4 School of Biomedical Engineering and Imaging Sciences, King's College London, London, UK; 5 Big Data Institute, University of Oxford, Oxford, UK; 6 Department of Clinical Neurosciences, University of Lausanne, Lausanne, Switzerland; 7 Department of Psychology, University of Oslo, Oslo, Norway; 8 Dementia Research Centre, Institute of Neurology, University College London, London, UK; 9 Donders Centre for Cognition, Donders Institute for Brain, Cognition and Behaviour, Radboud Universiteit, Nijmegen, The Netherlands; 10 Inserm U1153, Epidemiology of Ageing and Neurodegenerative diseases, Université Paris Cité, Paris, France; 11 Faculty of Brain Sciences, University College London, London, UK

**Keywords:** PSYCHIATRY, Delirium & cognitive disorders, Adult psychiatry

## Abstract

**Background:**

Current dementia risk scores have had limited success in consistently identifying at-risk individuals across different ages and geographical locations.

**Objective:**

We aimed to develop and validate a novel dementia risk score for a midlife UK population, using two cohorts: the UK Biobank, and UK Whitehall II study.

**Methods:**

We divided the UK Biobank cohort into a training (n=176 611, 80%) and test sample (n=44 151, 20%) and used the Whitehall II cohort (n=2934) for external validation. We used the Cox LASSO regression to select the strongest predictors of incident dementia from 28 candidate predictors and then developed the risk score using competing risk regression.

**Findings:**

Our risk score, termed the UK Biobank Dementia Risk Score (UKBDRS), consisted of age, education, parental history of dementia, material deprivation, a history of diabetes, stroke, depression, hypertension, high cholesterol, household occupancy, and sex. The score had a strong discrimination accuracy in the UK Biobank test sample (area under the curve (AUC) 0.8, 95% CI 0.78 to 0.82) and in the Whitehall cohort (AUC 0.77, 95% CI 0.72 to 0.81). The UKBDRS also significantly outperformed three other widely used dementia risk scores: the Australian National University Alzheimer’s Disease Risk Index (ANU-ADRI), the Cardiovascular Risk Factors, Ageing, and Dementia score (CAIDE), and the Dementia Risk Score (DRS).

**Clinical implications:**

Our risk score represents an easy-to-use tool to identify individuals at risk for dementia in the UK. Further research is required to determine the validity of this score in other populations.

**WHAT IS ALREADY KNOWN ON THIS TOPIC**

- Current dementia risk scores have limited generalisability.

**WHAT THIS STUDY ADDS**

- We have developed the UK Biobank Dementia Risk Score (UKBDRS), a dementia risk score based on large UK-based cohorts which outperforms three commonly used scores.

**HOW THIS STUDY MIGHT AFFECT RESEARCH, PRACTICE OR POLICY**

- The UKBDRS represents a scalable tool for dementia risk stratification in the UK. Our results suggest caution when applying risk scores across different populations.

## Background

 An estimated 50 million individuals are currently living with dementia worldwide.^1^ With the number of dementia cases projected to triple by 2050, prevention is a crucial avenue for addressing this public health challenge.^1^ Up to 40% of dementia cases may be prevented by targeting 12 key risk factors, including low education levels, smoking, hypertension, obesity, diabetes and excessive alcohol intake.^2^ Prognostic models of dementia risk which incorporate these factors may help identify high-risk individuals while they are still in the prodromal phases and direct them towards interventions to delay or prevent dementia.^3^

Several prognostic models have been developed to predict individual-level dementia risk. These models vary in the factors used to predict risk, and have previously included various combinations of sociodemographic, cognitive, imaging, biomedical and genetic variables.^4^ However, while the availability and use of dementia risk scores is increasing, there are many limitations which still need to be addressed.^5^

For example, risk scores are often developed in a single population without external validation. While increased attention to this issue has been paid in recent years,^6 7^ problems with external validation still persist. For instance, a 2019 systematic review of 61 available dementia risk scores found that only eight had been externally validated.^3^ Moreover, those that had been validated often had poor and inconsistent performance in external samples. An external validation of four commonly used prediction models in a Dutch population found that only one model performed strongly (c-statistic >0.8) and all models were poorly calibrated with underestimation of low risk and overestimation of higher risk.^6^ A recent large-scale validation study assessed the performance of 17 risk models in an Icelandic population and found that only models which included comprehensive cognitive testing achieved moderate prediction accuracy (c-statistic >0.75), while all other models performed poorly (c-statistic <0.75).^8^

Several reasons may underlie the poor out-of-sample performance of dementia risk scores. First, the factors associated with dementia, as well the importance of each factor, may vary across different geographical locations. Most development cohorts are typically from North America, with few others coming from across Europe.^3 8^ To our knowledge, there is only one dementia risk model that has been developed for the UK population, and little is known about how other externally-developed dementia risk models may perform in the UK.^9^ Second, the risk factors associated with dementia may vary across the lifespan. Interestingly, Hou *et al* noted that only four risk scores have been developed for use in mid-life compared with 39 developed for use in elderly populations.^3^ Thus, many of the available models may not be applicable to adults in mid-life who stand to benefit the most from early interventions. Third, model overfitting could underlie poor out-of-sample performance, particularly as, until recently, the standard approach for developing risk scores involved conducting numerous univariate models to identify the best predictors of dementia from a long list of candidate predictors. Many dementia risk scores may integrate invasive (cerebrospinal fluid), time-intensive (cognitive batteries), or expensive (MRI) markers. While such scores may offer modest improvements of prediction accuracy, they are more appropriate for specialised clinical settings rather than routine primary care or for population screening where these measurements are not available at scale.^5^

### Objectives

In this study, we address these limitations by using data from the UK Biobank (UKB) study to develop a novel 14-year dementia risk score specific to a mid-life UK population, which we refer to as the UK Biobank Dementia Risk Score (UKBDRS). We externally validate our score in the Whitehall II (WHII) study, another UK-based cohort with mid-life assessments. We focus on easily accessible sociodemographic and clinical data to enable the wider use of the score for routine care or large-scale screening for preventative trials. To combat the challenge of overfitting, we employ regularised regression methods to select variables used in the UKBDRS. We also compare the performance of our score to three previously published dementia risk scores: the Australian National University Alzheimer’s Disease Risk Index, ANU-ADRI^10^, the Cardiovascular Risk Factors, Ageing, and Dementia score, CAIDE^11^, and the Dementia Risk Score, DRS^9^. We chose these other scores as they are widely used in research studies worldwide, have previously been validated in at least one external cohort,^3 6^ are composed of easily accessible risk factors, and therefore offer a reasonable comparative framework against which to test our novel score.

## Methods

### Sample selection

The UKBDRS was developed and validated internally in the UKB study and tested externally in the WHII study cohort. The UKB^12^ is a longitudinal cohort study of over 500 000 individuals (age range 40–73 years). The WHII cohort^13^ includes 10 308 British civil servants recruited in 1985 (age range 35–55 years) who have since received comprehensive clinical examinations approximately every 5 years across 12 study waves. In both samples, we excluded individuals younger than 50 years in order to focus our analysis on those with increased likelihood of developing dementia within the study follow-up period, and to reduce the likelihood of including those with monogenic risk of developing dementia. We also excluded individuals with missing data on any of the candidate risk factors described. For detailed sample selection and inclusion/exclusion criteria, please see online supplemental figure 1 and the online supplemental methods. The UKB sample was split into a training set (80% of sample) and test set (20%), stratified for proportion of incident cases. APOE genotype is a strong, but less readily available, risk factor. Thus, we performed a second analysis to compute a version of the UKBDRS including APOE genotype.

The UKB study received ethical approval from the Northwest Multi-Centre Research Ethics Committee and the WHII study obtained ethical approval from the University College London Medical School Committee on the Ethics of Human Research. All participants provided their informed written consent.

### Dementia ascertainment

In the UKB sample, all-cause dementia status was determined based on complementary sources of information as done in several papers based on this cohort.^14 15^ An individual was classified as having dementia if they had either (1) self-reported a diagnosis at baseline (excluded from analyses), (2) received a primary or secondary diagnosis of dementia (primary care/hospital records), (3) were prescribed dementia-related medications (eg, rivastigmine) by their general practitioner, or (4) if their primary or secondary cause of death was dementia-related. UKB field IDs used for diagnosis and the list of International Classification of Disease ninth revision (ICD-9) and ICD 10th revision (ICD-10) codes are presented in the online supplemental table 1. In the WHII sample, dementia diagnosis was determined through self-report and hospital inpatient records^16 17^ (online supplemental methods, and online supplemental table 1).

### Identifying candidate predictors

We compiled a list of 28 risk and protective factors associated with dementia, including 11 of the 12 modifiable factors identified by the Lancet Commission.^2^ Predictors were selected for inclusion if (1) they had been consistently associated with dementia, (2) information about these was available in UKB, and (3) they could be easily obtained within a primary care setting. The full list of predictors’ detailed descriptions including UKB field codes are in online supplemental methods and online supplemental table 2. All predictors were measured at baseline.

### Construction of existing risk scores

The predictive ability of the UKBDRS was compared with that of three existing risk scores: the DRS,^9^ ANU-ADRI,^10^ and CAIDE.^11^ We computed the three risk scores in the UKB and WHII cohorts using the formulae reported in the respective original papers (online supplemental methods).

### Statistical analyses: development of the UKBDRS

All continuous variables were standardised and outliers (ie, individuals with values <Q1 − 3×IQR or values >Q3 + 3×IQR) were excluded (1.8% of dataset). The first stage (‘variable selection’) used only the training set and involved submitting the 28 candidate predictors to a least absolute shrinkage and selection operator (LASSO) Cox regression (online supplemental methods), to identify a parsimonious model with dementia as the outcome.^18^ Correlation between numerical variables was assessed before LASSO (online supplemental methods, online supplemental figure 2). LASSO selected variables were then used as predictors in a Fine and Gray competing risk regression model.^19^ Duration of follow-up was calculated as time between baseline and either date of dementia, death, or censoring date (online supplemental methods). The linear predictor was used to compute the predicted probability of developing dementia (online supplemental methods). Two variants of the UKBDRS were derived: a score without APOE (UKBDRS) and a score with APOE (UKBDRS-APOE). Our primary model is the UKBDRS, as genetic information may not be widely available. Assumptions were checked as described in online supplemental methods (online supplemental figures 3 and 4).

### Statistical analyses: evaluation of the UKBDRS

The performance of the UKBDRS was compared with the DRS, CAIDE, and ANU-ADRI in the UKB train, test and WHII datasets. All models were additionally compared with a baseline model consisting of chronological age only, to examine the added predictive value of additional factors. The model discrimination was evaluated using the area under the curve (AUC). Based on available follow-up times, we evaluated AUC at a 14-year time horizon in UKB and 17-year in WHII (online supplemental methods). Wald’s test^20^ was used for pairwise comparisons of the AUCs of the UKBDRS with the other risk models. P values were corrected for multiple comparisons (false discovery rate (FDR) p value ≤0.05 was considered significant).

We used risk calibration to assess the agreement between the observed proportion of dementia cases and predicted probabilities of developing dementia as calculated from the risk score^21^ (online supplemental methods). This study is reported in accordance with the Transparent Reporting of a Multivariable Prediction Model for Individual Prognosis or Diagnosis (TRIPOD) statement. Data analysis was conducted in R (3.6.3) and Python (3.6), using FSL’s funpack and using the following R packages: CARET,^22^ glmnet,^23^ cmprsk,^19^ survival,^24^ and riskRegression.^20^

### Sensitivity analyses

We also evaluated performance of each score only in the age ranges for which the external scores were originally developed. To do this we truncated the UKB test and WHII cohorts to match the age range of original development and validation cohorts and computed the AUC. We also assessed performance of the score at 5-year and 10-year time windows.

### Data and code availability

The data used in this study are available from the UK Biobank (https://www.ukbiobank.ac.uk/enable-your-research/apply-for-access) and DPUK portal (https://portal.dementiasplatform.uk/Apply). As restrictions apply to the availability of these data, which were used under licence for the current study, the authors cannot publicly share these data. This research has been conducted using the UK Biobank Resource under Application Number 47279, while the use of data from the WHII study was approved by the DPUK Access Committee (Project No. 0346). Code is available at https://github.com/MelisAnaturk/dementia_risk_score.

## Findings

### Sample characteristics

Totals of 220 762 (mean age 59.97 years, SD 5.43) and 2934 (median age 57, IQR 10) individuals were in the analysis samples for UKB and WHII, respectively. The sample characteristics are in online supplemental table 3, and participant selection is in online supplemental figure 1; 3813 (1.7%) and 93 (3.2%) participants developed dementia in the UKB and WHII cohorts, respectively. Our score represents the predicted probability of developing dementia in 14 years. The maximum years to diagnosis in UKB was 14.2, and thus 14 years was used as the time window for dementia prediction in UKB. There were significant differences in age, sex, and education between included and excluded participants in UKB and WHII (online supplemental table 4). The UKBDRS-APOE was computed on subsets with complete APOE information (n=157 090 for UKB and n=2315 for WHII).

### Selection of predictors for the UKBDRS

LASSO regression identified 11 variables as predictive of incident dementia: age, education, history of diabetes, history/current depression, history of stroke, parental history of dementia, Townsend deprivation, hypertension, high cholesterol, household occupancy (living alone), and sex (online supplemental table 5). The beta coefficients for the final competing risk regression models are provided in table 1.

**Table 1:** Results of the competing risk regressions with two variants of the UKBDRS

Predictor	β	HR	95% CI Lower Upper		P
UKBDRS					
Age (years)	0.178	1.194	1.184	1.206	2.1×10^−296^
Parental history (yes)	0.431	1.539	1.415	1.674	2.1×10^−296^
Education (years)	−0.041	0.960	0.948	0.972	2.1×10^−296^
Townsend deprivation (most deprived)	0.228	1.256	1.153	1.367	2.1×10^−296^
Diabetes (yes)	0.536	1.710	1.528	1.914	2.1×10^−296^
Depression (yes)	0.556	1.744	1.593	1.909	2.1×10^−296^
Stroke (yes)	0.655	1.925	1.652	2.242	2.1×10^−296^
Hypertensive (yes)	0.159	1.173	1.082	1.271	2.1×10^−296^
High cholesterol (yes)	0.104	1.110	1.015	1.213	2.1×10^−296^
Sex (male)	0.169	1.184	1.099	1.275	2.3×10^−2^
Lives alone (yes)	0.141	1.151	1.058	1.253	1×10^−3^
UKBDRS-APOE					
Age (years)	0.185	1.204	1.191	1.217	2.1×10^−296^
Parental history (yes)	0.311	1.365	1.24	1.503	2.1×10^−296^
Education (years)	−0.038	0.963	0.949	0.977	2.1×10^−296^
Townsend deprivation (most deprived)	0.247	1.28	1.16	1.412	2.1×10^−296^
Diabetes (yes)	0.526	1.692	1.479	1.936	2.1×10^−296^
Depression (yes)	0.567	1.763	1.587	1.959	2.1×10^−296^
Stroke (yes)	0.643	1.902	1.586	2.28	2.1×10^−296^
Hypertensive (yes)	0.190	1.209	1.101	1.326	2.1×10^−296^
High cholesterol (yes)	0.027	1.028	0.928	1.138	6×10^−1^
Sex (male)	0.164	1.179	1.082	1.284	7×10^−3^
Lives alone (yes)	0.137	1.146	1.039	1.264	2.1×10^−296^
APOE4 genotype (yes)	1.129	3.091	2.845	3.359	2.1×10^−296^

For each predictor, the β coefficient and hazard ratio (HR) are presented, along with the 95% confidence interval of the HR and the Bonferroni-Holmes corrected p value. 14-year baseline survival, $S_{0}$, is 0.9916195 for the UKBDRS model and 0.9945524 for the UKBDRS-APOE model.

UKBDRS, UK Biobank Dementia Risk Score.

### Discrimination and calibration in the UKB and WHII test sets

In the UKB test sample, the AUC of the UKBDRS was 0.8 (95% CI 0.78 to 0.82), while the UKBDRS-APOE achieved an AUC of 0.83 (95% CI 0.81 to 0.84) in the subset of UKB individuals with available genotype data. In the WHII sample, AUC of the UKBDRS was 0.77 (95% CI 0.72 to 0.81), and AUC of the UKBDRS-APOE was 0.79 (95% CI 0.74 to 0.85) (table 2). Sensitivity analyses showed that the UKBDRS performed better in predicting dementia within 14 years compared with prediction of dementia within shorter time horizons of 5 years (AUC 0.75, 95% CI 0.71 to 0.78) or 10 years (AUC 0.76, 95% CI 0.74 to 0.78) (online supplemental table 6).

**Table 2:** Discrimination accuracy of models across the training and test sets

	UKB train	UKB test	WHII
N	176 611	44 151	2934
Baseline model			
Age only	0.75 (0.75 to 0.75)	0.77 (0.75 to 0.79)	0.74 (0.69 to 0.78)
UKBDRS			
UKBDRS	0.79 (0.78 to 0.79)	0.80 (0.78 to 0.82)	0.77 (0.72 to 0.81)
UKBDRS-APOE	0.81 (0.81 to 0.81)	0.83 (0.81 to 0.84)	0.80 (0.75 to 0.85)
Other risk scores			
CAIDE	0.60 (0.60 to 0.60)	0.60 (0.58 to 0.63)	0.69 (0.64 to 0.74)
DRS	0.76 (0.76 to 0.76)	0.77 (0.76 to 0.79)	0.74 (0.69 to 0.78)
ANU-ADRI	0.57 (0.57 to 0.57)	0.57 (0.54 to 0.59)	0.52 (0.45 to 0.58)

AUCs are reported with 95% confidence intervals indicated in parentheses. 0.9% of the UKB sample had missing data for one variable of the ANU-ADRI score (BMI). Therefore, all individuals with missing data on the ANU-ADRI were first excluded before evaluating the AUC for the ANU-ADRI.

ANU-ADRI, Australian National University Alzheimer’s Disease Risk Index; AUC, area under the curve ; BMI, body mass index; CAIDE, Cardiovascular Risk Factors, Aging and Dementia; DRS, Dementia Risk Score; UKB, UK Biobank; UKBDRS, UK Biobank Dementia Risk Score; WHII, Whitehall II.

**Figure 1:**
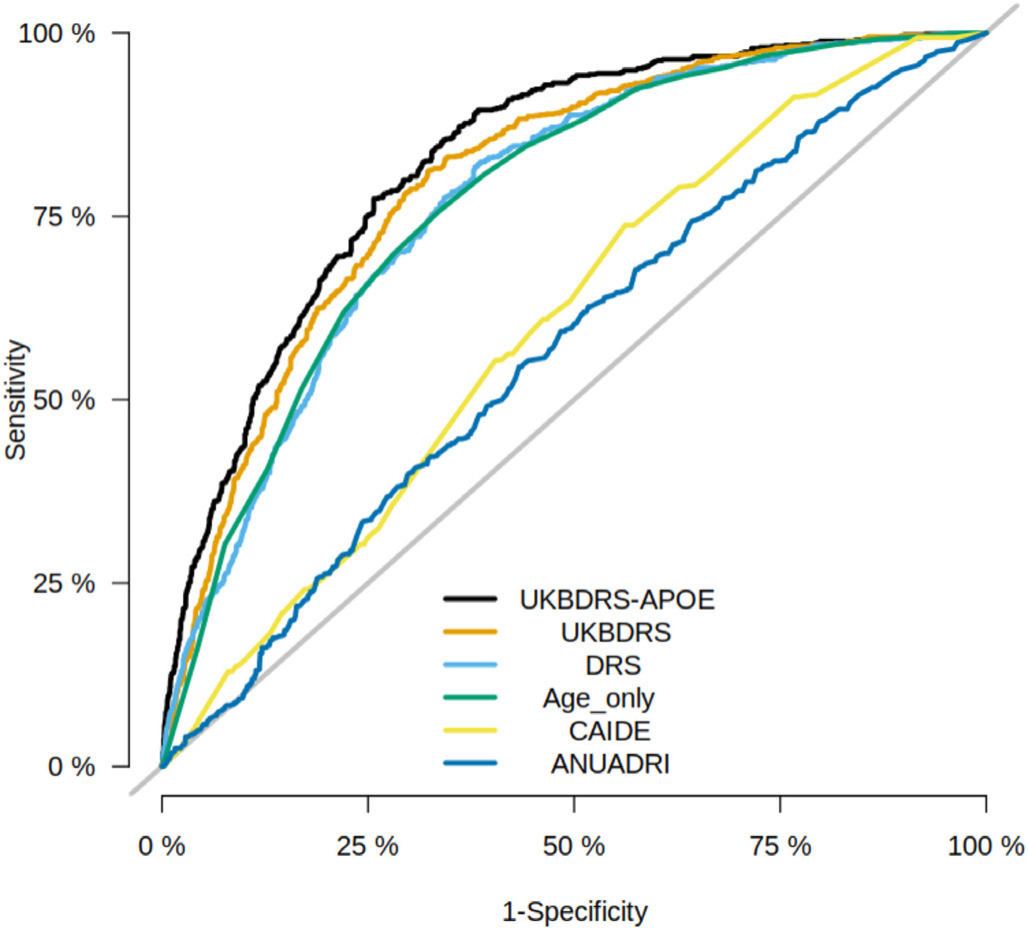
Receiver-operating characteristic curves (ROC) for each risk score in the UKB test set. Sensitivity and specificity (plotted from 1 to 0) at varying thresholds to build and ROC curve for each risk score computed in the UKB test set. The highest performing score, as indicated by the greatest area under the curve, was the UKBDRS-APOE model, followed by the UKBDRS, age only, DRS, CAIDE, and ANU-ADRI. The UKBDRS-APOE performance is assessed in the subset of individuals with genotype information available. ANU-ADRI, Australian National University Alzheimer’s Disease Risk Index; CAIDE, Cardiovascular Risk Factors, Aging and Dementia; DRS, Dementia Risk Score; UKB, UK Biobank; UKBDRS, UK Biobank Dementia Risk Score; WHII, Whitehall II.

In both the UKB test sample and the external WHII validation, the UKBDRS had significantly higher AUCs compared with the age-only model, the DRS, CAIDE, and the ANU-ADRI (p_corr_<0.05, table 3). Figure 1 plots the ROC curves for all models. In sensitivity analyses conducted in the UKB training and test samples, the UKBDRS consistently outperformed external risk models even when we restricted the analysis to the age range for which each external score was originally developed. In sensitivity analyses conducted in the WHII sample, the UKBDRS model outperformed CAIDE but performed similarly to the DRS and ANU-ADRI in individuals over 60 (online supplemental table 7). Online supplemental table 8 presents sensitivity, specificity, negative predictive value (NPV) and positive predictive value (PPV) of both UKBDRS scores at various thresholds. We have also presented these data as a UKBDRS Calculator, an excel sheet where users can enter responses for the 11 measures of UKBDRS and receive a 14-year risk of dementia (https://github.com/MelisAnaturk/dementia_risk_score/blob/main/results/UKB-DRS_Calculator.xlsx). UKBDRS models were well-calibrated—that is, they had an intercept and slope close to 0 and 1, respectively (online supplemental table 9 and figure 5).

**Table 3:** Pairwise comparisons in the UKB test set and WHII validation sample to survive FDR corrections

Risk score 1	AUC for risk score 1	Risk score 2	AUC for risk score 2	P	P_corr_
UKB					
UKBDRS	0.80	Age only	0.77	9.54×10^-6^	9.54×10^-6^
UKBDRS	0.80	ANU-ADRI	0.57	2.62×10^-62^	1.05×10^-61^
UKBDRS	0.80	CAIDE	0.60	2.23×10^-42^	4.45×10^-42^
UKBDRS	0.80	DRS	0.77	5.64×10^-7^	7.53×10^-7^
WHII					
UKBDRS	0.77	Age only	0.74	3.20×10^-3^	5.97×10^-3^
UKBDRS	0.77	ANU-ADRI	0.52	8.16×10^-11^	3.27×10^-10^
UKBDRS	0.77	CAIDE	0.69	4.48×10^-3^	5.97×10^-3^
UKBDRS	0.77	DRS	0.74	1.07×10^-2^	1.07×10^-2^

.The AUC of the UKBDRS was compared with each external risk score and an age-only model. If a comparison is not shown, there was no statistical difference in performance.

ANU-ADRI, Australian National University Alzheimer’s Disease Risk Index; AUC, area under the curve; CAIDE, Cardiovascular Risk Factors, Aging and Dementia; DRS, Dementia Risk Score; FDR, false discovery rate; UKB, UK Biobank; UKBDRS, UK Biobank Dementia Risk Score; WHII, Whitehall II.

## Discussion

We developed a risk score for predicting up to 14-year all-cause incident dementia in individuals 50–73 years old, and evaluated it against three published risk scores.^9^,^11^ A model consisting of age, education, diabetes, depression, stroke, parental history of dementia, material deprivation, hypertensive status, cholesterol status, sex, and household occupancy achieved a good-to-strong predictive accuracy and was well calibrated, with and without APOE4. Importantly, the discriminative performance of our score was consistently higher than three other established risk models, further supporting its utility in predicting individual-level risk of dementia in a mid-life UK population. We offer an excel sheet (https://github.com/MelisAnaturk/dementia_risk_score/blob/main/results/UKB-DRS_Calculator.xlsx) to calculate an individual’s risk of dementia (we stress that this tool is for illustrative purposes only, not for clinical use, and does not replace clinical judgement). Individuals can also be classified into low-risk and high-risk groups, according to sensitivity and specificity thresholds reported in online supplemental table 9. As the information required for computing the UKBDRS can be easily collected at a population level, and some can be managed by targeted interventions, the UKBDRS may be a promising screening tool for stratifying middle-aged UK individuals for preventative interventions. Our study also raised important considerations about the reliability of dementia risk scores, as evidenced by the poor out-of-sample performance of some external scores. We therefore discuss recommendations for using dementia risk scores in clinical trials.

The UKBDRS includes established modifiable risk factors for dementia, namely stroke, type 2 diabetes, hypertension, and depression.^25^ Our score also included material deprivation, fewer years of education, parental history, and living alone (ie, social isolation) as markers of increased risk, all of which have been repeatedly associated with dementia.^2 26 27^

In contrast to other risk scores, our model identified male sex as a predictive factor for dementia. Interestingly, a recent study of the UKB found that the risk of dementia was higher in men than women, and that a greater proportion of men had impaired cardiovascular health.^14^ Thus, our findings are in line with other studies of the same population and may be driven by underlying cardiovascular issues. Our model also did not identify other factors previously linked to dementia risk, such as body mass index (BMI) and physical inactivity.^2 3^ It is possible that even if these lifestyle factors have been linked to risk of dementia, (1) their predictive utility for dementia may change with age and time to diagnosis, and (2) their non-specific role in the development of other diseases in later life may lessen their sensitivity and specificity for dementia. It is important to note that the goal of our study was to create an accurate, parsimonious model, as opposed to identifying all factors associated with dementia.

The UKBDRS consistently outperformed three scores previously developed for dementia risk prediction (ie, DRS, ANU-ADRI, CAIDE) in both internal and external validation samples. Our AUCs for the external dementia scores fell within the range of values reported from previous validation attempts (CAIDE 0.49–0.78^6 7 11^; ANU-ADRI 0.49–0.78^6 7 10^; DRS 0.56–0.84^6 9^). Both ANU-ADRI and CAIDE also performed worse in comparison to a model based solely on age, a striking observation but in line with their recent evaluation in the Rotterdam cohort.^6^ This could be for several reasons. First, certain predictors may not map precisely from one dataset to another. For example, CAIDE defines physically active individuals as those who exercise at least twice a week, lasting at least 20–30 min each time. UKB participants were instead asked how many minutes were spent being physically active on a typical day. Second, ANU-ADRI was developed for older individuals (60+), while our cohort is slightly younger (50+). However, our sensitivity analysis found that the ANU-ADRI also performed poorly when restricting our cohort to an age range matching its development sample. Third, CAIDE was designed to predict dementia over 20 years, while our cohorts had timeframes of only 14 and 17 years, respectively. Fourth, there were differences in diagnosis procedures between cohorts. CAIDE performed diagnosis according to DSM designations. Similarly, ANU-ADRI was developed specifically for Alzheimer’s disease as opposed to the all-cause dementia outcome in our study. Finally, despite including all the predictors from the CAIDE and ANU-ADRI (except pesticide exposure) in our initial LASSO regression, only four of the seven predictors from CAIDE and four of the 12 predictors from ANU-ADRI were identified as important predictors in UKB. Five of the 14 predictors from the DRS model were initially selected as relevant predictors by our LASSO model. Thus, the performance of risk scores may be driven by population-specific differences.

Our score included age, which may be questioned as it is not a modifiable target. However, if the goal is to stratify for risk, then the inclusion of age is obvious. In this context, we note the good performance of the baseline age-only model. Age may proxy relevant information from several age-associated medical predictors (eg, cardiovascular health). A recently developed risk score which explicitly excluded age to focus on modifiable factors achieved an AUC of 0.59, demonstrating that age can be expected to be the driving force in many risk scores and poor discrimination can be expected when not considering age.^28^ We therefore suggest that risk scores with the explicit goal of risk stratification include age and other non-modifiable factors to achieve a stronger predictive performance.

It is possible our score could be improved by adding cognitive tests, brain MRI, and blood-based biomarkers of neurodegeneration.^5^ A recent review noted that high-performing risk scores included these variables, but as they are expensive and/or time consuming they would have limited application in population-level settings.^3^ Moreover, UKB uses cohort-specific cognitive tests rather than established cognitive batteries typically used for dementia screenings which would have made it difficult to validate a cognition-based UKB score in other cohorts that do not have similar cognitive measures. Therefore, the UKBDRS may best be used as an initial screening tool to stratify individuals into risk groups, and those identified as high risk could then benefit from more time-consuming follow-up assessments described above for more detailed characterisation.

Several limitations need to be considered when interpreting our findings. First, we note that the UK Biobank does not have a gold standard clinically adjudicated dementia diagnosis. Nonetheless, our approach, which used a combination of primary care, hospital inpatient records, death certificates, and self-report, has previously demonstrated a strong PPV of 82.5% when compared with clinical adjudication,^29^ and has been used extensively in the UKB cohort.^14 15^ As the reported PPV of this approach is lower when stratifying outcomes by dementia sub-type, we restricted our outcome to all-cause dementia.^29^

Second, the UKB and WHII cohorts differed in the availability of data used for dementia classification, which may partly explain the difference in the prevalence of all-cause dementia (1.7% in UKB vs 3.2% in WHII). While death, primary care and hospital records were supplemented by self-report in UKB, only hospital inpatient records and self-report were available for the WHII which may have affected how sensitive we were in identifying ‘true positives’. We also note cohort differences in demographics, lifestyle, and health variables which may partly explain the lower AUC achieved in the external WHII sample.

Third, there were differences in the prevalence of factors used to compute the external scores, potentially affecting their predictive ability. For example, the UKB version of the ANU-ADRI excluded the cognitive component while the WHII version of this score missed information on traumatic brain injury and pesticide exposure. Moreover, WHII participants consumed more units of alcohol per week relative to UKB (18.8 units/week vs 14.3 units/week) and had substantially fewer women (28% vs 51%). The UKB also does not offer information on whether participants need help with their finances or medications, which prevented us from computing other risk models. We used complete case analyses and did not impute missing data to mimic settings in clinical practice. This may have introduced certain biases in our results.

Fourth, it is possible the samples of UKB and WHII data assessed introduced a healthy volunteer bias. While there were statistically significant differences between the included and excluded participants in terms of age, sex, and education, these were small (eg, age: 59.97 vs 60.71). The UKB and WHII cohorts are also healthier than the general population. For example, the prevalence of risk factors (eg, BMI, smoking) and rates of disease in the UKB are lower than in the general population.^30^ We used electronic health records to derive diagnoses, which make this large sample analysis feasible but may be less sensitive than clinical adjudication. Notably, the incidence of dementia in the UKB and WHII is lower than values reported for European and American populations and therefore under-ascertainment of dementia may be a limitation. Both the UKB and WHII cohorts are predominantly Caucasian and are less likely to live in socioeconomically deprived areas than the general UK population.^13 30^ It is well known that dementia risk, onset and prevalence vary by race, ethnicity and socioeconomic status.^7^ Therefore, while the consistent performance of UKBDRS across these two independent cohorts adds confidence to its robustness, we emphasise the need to evaluate it across more diverse cohorts both in and out of the UK before translating it for wider use.

### Clinical implications

We have developed and validated a novel dementia risk score, which outperformed other established risk scores in both an internal and external validation dataset. We recommend the UKBDRS for future studies interested in midlife dementia risk in a UK population. The UKBDRS includes age, education, material deprivation, history of diabetes, depression, stroke, parental history of dementia, hypertensive status, cholesterol status, sex, and household occupancy, making it easy to assess in large populations. We also present a version of the UKBDRS which includes APOE4 and can offer improved performance if this information is available. With further validation, the UKBDRS may be useful as a dementia screening tool for a wide range of middle-aged adults in either a clinical or research setting.

Importantly, this study raises concerns about the overall generalisability of dementia risk models. Our findings and others have shown that dementia risk scores typically have reduced predictive accuracy when applied under different settings. This suggests that there is unlikely to be a single ‘one-size-fits-all’ dementia risk score for all populations. Instead, we suggest that any clinical trial should carefully consider the demographics of their participants and either identify a closely matched risk score, or pool together predictions from multiple risk models, with the UKBDRS potentially serving as one of these models.

## Supplementary material

10.1136/bmjment-2023-300719 online supplemental file 1
