# Supplementary material for: Development and validation of a dementia risk score in the UK Biobank and Whitehall II cohorts
Source: BMJ Ment Health. 2023 Aug 21;26(1):e300719. doi: 10.1136/bmjment-2023-300719 (PMC10577770; doi:10.1136/bmjment-2023-300719)
Supplement: online supplemental file 1 [file bmjment-2023-300719supp001.pdf]

Supplementary Information - Methods

Sample Selection

The UK Biobank (UKB) [1] is a longitudinal cohort study of over 500,000 individuals (age range: 40-69). At their baseline visit (2006-2010), participants attended one of 22 sites across the UK to undergo several assessments, including an interview covering their pre-existing health conditions, self-report questionnaires, biomedical assessments, and collection of blood, urine, and saliva samples. Approximately 230,000 (45% of sample) participants have provided consent for UKB to access their National Health Services (NHS) health records. This is supplemented by linkage to inpatient hospital admissions (http://biobank.ndph.ox.ac.uk/showcase/ refer.cgi?id=138483) and national death registries (http://biobank.ctsu.ox.ac.uk/crystal/refer.cgi?id=115559).

The WHII cohort [2] includes 10,308 British civil servants recruited in 1985 (age range: 35-55) who have since received comprehensive clinical examinations approximately every five years across 12 study waves. Along with its extensive follow-up period, this cohort has a detailed set of lifestyle-, biological, and health-related measures. Most participants are now >65 years old and some have received a diagnosis of dementia [3].

Inclusion and Exclusion Criteria

For the UKB study, we restricted our sample to participants aged 50+, and who had complete data available on all candidate predictors of interest, described in section 2.3. To improve alignment of age ranges between the WHII and UKB samples, Wave 5 (1997-1999) of the WHII study was selected as our ‘baseline’, given that the minimum age of the sample at that Wave was just above 40. To minimise the likelihood of reverse causation, we excluded participants who self-reported dementia at baseline, or developed dementia prior to or within the first 12 months of the baseline assessment in UKB. Similarly, for WHII, we excluded participants who self-reported a diagnosis at Wave 6 (2001, median years after baseline = 3), or anyone who had developed dementia within 2 years of the baseline visit. The two-year criterion was introduced to ensure that at least 12 months had passed between the baseline assessment and diagnosis date, given that the WHII sample only had the calendar year of dementia diagnosis available. For example, this would prevent including someone who had attended their Wave 5 appointment in December 1997 and then received a dementia diagnosis in January 1998. Individuals in the WHII cohort with no recorded follow up (i.e. attended baseline assessment and no further assessment, or no date recorded at later assessment) were also excluded due to inability to determine length of follow up time.

Dementia Ascertainment

In the UKB sample, all-cause dementia status was determined based on complementary sources of information as done in several papers based on this cohort [4–10]. This included self-reported medical history at the time of interview (UKB field ID #20002), primary care records (#42040, #42039), hospital inpatient records (#41270, #41280) and death registry records

(#40001, #40002). The International Classification of Diseases (ICD) was used to identify cases with all-cause dementia, with the list of ICD-9 and ICD-10 codes presented in the SI Table 1. ICD-9 codes were included to enhance our sensitivity to detecting positive cases of dementia prior to enrolment. In the UKB cohort, an individual was classified as having dementia if they had either (1) self-reported a diagnosis at baseline (excluded from analyses), (2) received a primary or secondary diagnosis of dementia (primary care/ hospital records), (3) were prescribed dementia-related medications (e.g., rivastigmine) by their general practitioner (GP), or (4) if their primary or secondary cause of death was dementia-related. For individuals with multiple dates available for the above variables, the date of diagnosis was either determined with algorithmically defined dates (field ID #42018), or otherwise the date of a primary care diagnosis, primary care prescription date for dementia-related medications or date of death derived from primary care records, whichever date came earliest. Years to diagnosis was then calculated as the difference between the baseline assessment date and the earliest date of dementia diagnosis. In the WHII sample, dementia diagnosis was determined through self-report of a long standing illness of dementia and hospital inpatient records [11–13] (SI Table 1). Date of diagnosis was taken as the earlier date of the two sources. For those with only a self report of long standing illness of dementia reported at a study assessment, the date was taken as 1 year prior to the assessment year to account for lag in assessment.

Follow up and censoring derivation

In the UKB, the censoring date was 2021-10-31 which was the date until which diagnoses were available. Death records were used to obtain the date of death. For individuals with dementia, time at risk was calculated as the time in days between their baseline assessment and dementia diagnosis. For individuals with a death record and no diagnosis of dementia, time at risk was computed as the time between their baseline assessment and the date of their death record. For individuals alive and dementia free, time was taken as the time between their baseline assessment and the censoring date.

In the WHII, a combination of hospital records and study assessments were used to derive follow up. The latest assessment record was the later of an individual's last assessment date and the year of their most recent hospital admission. For example, an individual with a baseline assessment in 1997 who last attended a study wave in 2012 and had a hospital record in 2013 was defined as having a time at risk of 2013 - 1997 = 16 years. Date of death was drawn from the national mortality register.

Identifying Candidate Predictors

We compiled a list of 28 risk and protective factors associated with dementia, including the 12 modifiable factors identified by the Lancet Commission [14]. Predictors were selected for inclusion if (1) they had been consistently associated with dementia, (2) if information about these was available in UKB, and (3) they could be easily obtained within a primary care setting. The list included demographic, biomedical, lifestyle, and genetic variables. Demographic variables consisted of age, sex, years of education, and material deprivation. Material deprivation was measured by the Townsend Deprivation index, which combines data on car

ownership, household overcrowding, owner occupation, and unemployment to obtain a measure of material deprivation [36]. In the UK Biobank, scores are available as quintile splits, wherein they are computed for each postcode, then split into quintiles with the first quintile representing the fifth of the sample that was least deprived. In the WHII cohort, Townsend scores are available as quartile splits. Where applicable, we mapped the four quartiles in WHII to be the second to fifth quintiles as assessed in the UK Biobank dataset. Biomedical variables included body mass index (BMI), systolic blood pressure (BP), total cholesterol, high-density lipoprotein (HDL) cholesterol, and low-density lipoprotein (LDL) cholesterol. Medical history included self-reported history of a stroke/ transient ischaemic attack (TIA), traumatic brain injury (TBI), depression, diabetes (I and II), atrial fibrillation, hypertensive status, and high cholesterol and parental history of dementia. Hypertensive and cholesterol status were determined based on a combination of self report, self reported use of medications (anti-hypertensive or statins, respectively), or an inpatient diagnosis (SI Table 2 for codes used). Self-reported prescriptions of the following medications were also included: non-steroidal anti-inflammatory drugs (excluding aspirin) and hormone replacement therapy medications. Lifestyle variables consisted of physical activity (low, moderate/high-intensity classifications based on the International Physical Activity Questionnaire [15]), social engagement (household occupancy as living alone, living with one other, or living with multiple people, and frequency of family and friend visits per week), weekly fish consumption, average daily sleep duration, sleeplessness, weekly units of alcohol (computed as in [16, 17], and smoker status. The full list of predictors is available with detailed descriptions including UKB field codes in SI Table 2.

Construction of external risk scores

Each score was calculated using the formulae reported in the original papers for the DRS, ANU-ADRI and CAIDE. As described in the main text, the UKB-DRS and the three external risk scores were computed in (1) the entire UKB Test and WHII samples (results presented in Table 2) and (2) in a stratified analysis where the UKB and WH-II samples were truncated to match the age ranges of the cohorts in which the external scores were originally developed (SI Table 7).

ANU-ADRI: This score was originally developed using an evidence-based medicine approach, in which risk factors are identified based on review of the literature [18]. A total of 15 dementia risk factors are included in the ANU-ADRI, including age, sex, educational level, BMI, diabetes, TBI, depressive symptoms, high cholesterol, cognitive activity levels, social engagement, smoking, alcohol consumption, physical activity, fish intake and pesticide exposure. We computed the ANU-ADRI as the weighted sum of the beta coefficients of these risk factors, as reported in Anstey et al. [18]. For the UKB cohort, the necessary cognitive information was not available to accurately estimate cognitive activity levels, hence, this variable was excluded from the ANU-ADRI calculation. In the WHII, no information was available on pesticide exposure or whether a participant had a history of TBI. Hence, these variables were not included in the risk score calculation. Previous external validations of this score have also left out items such as pesticide exposure due to lack of data availability [19]. As a result, we were only able to

compute a weighted sum rather than a predicted risk. Therefore, only discrimination (not calibration) was evaluated for the ANU-ADRI.

DRS: The DRS [20] was originally developed using data (N = 930,395) from The Health Improvement Network (THIN) and offers two versions, one for individuals aged between 60-79 years old (N = 800,013, mean age = 65.6 ± 6.08) and another for individuals aged between 80-95 years old (N = 130,382, mean age = 84.8 ± 3.93). Given the age range of the UKB and WHII, we opted to use the DRS for 60-79 year-old individuals for all analysis in this study. The DRS score is calculated using age, sex, BMI, calendar year (of participation), deprivation score, smoker status, drinker status, current depression and antidepressant use, aspirin use, history of stroke and TIA, history of atrial fibrillation and diabetes. While the deprivation score is computed using 5 equal groups based on quintiles of the Townsend deprivation index, only four groups based on deprivation scores were available in the WHII cohort. The DRS score is computed as: 0.20921 × (age − 65.608) − 0.00339 × (age − 65.608) × (age − 65.608) − 0.0616 × (BMI − 27.501) + 0.002508 × (BMI − 27.501) × (BMI− 27.501) + 0.12854 × (sex, female = 1) + 0.13199 × (hypertension) + 0.04477 × (current year − 2003.719) + 0.013371 × (deprivation quintile 2) + 0.117904 × (deprivation quintile 3) + 0.201776 × (deprivation quintile 4) + 0.225529 × (deprivation quintile 5) − 0.06792 × (former smoker) − 0.08657 × (current smoker) + 0.443535 × (heavy drinking) + 0.833612 × (current depression and/or use of antidepressants) + 0.252833 × (current aspirin use) + 0.577207 × (history of stroke or TIA) + 0.220728 × (history of atrial fibrillation) + 0.286701 × (history of diabetes). Dementia risk is then computed as:

$$P(dementia) = 1 - (0.9969)^{e^{DRSscore}}$$

CAIDE: The CAIDE [21] score was originally developed to predict 20-year all-cause dementia risk in a midlife cohort (Cardiovascular Risk Factors, Aging and Dementia cohort, N = 1,409, mean age = 50.4 ± 6, age range 39-64). The CAIDE score is computed by assigning points based on an individual's age (< 47 years: 0 points, 47–53 years: 3 points and > 53 years: 4 points), sex (men: 1 point), education (≥ 10 years: 0 points, 7–9 years: 2 points, 0–6 years: 3 points), hypertension (> 140 mmHg: 2 points), body mass index (> 30 kg/m2: 2 points), cholesterol (> 6.5 mmol/L: 2 points), and physical activity (inactivity: 1 point). Dementia risk is then computed via the following formula:

$$P(dementia) = \frac{e^{\left(\beta_0 + \beta_1 + \beta_2[score]\right)}}{1 + e^{\left(\beta_0 + \beta_1 + \beta_2[score]\right)}}$$

Where β_0 is the intercept (-7.406), β_1 is the coefficient of follow-up time (0.796), and β_2 is the CAIDE score.

Development of the UKBDRS

To identify a parsimonious model, we submitted the candidate predictors to a least absolute shrinkage and selection operator (LASSO) **cox** regression. Recently utilised in the development of dementia risk models [22, 23], LASSO is a type of regularised regression developed to minimise model overfitting [24], This method favours a sparse solution by setting the coefficients

of predictors that fall below a certain threshold to zero [24, 25], effectively providing a list of the most informative predictors of dementia. An initial ten-fold cross-validation was performed to identify the optimal lambda value that determines this threshold. The highest lambda within one standard error of the minimum was selected for further analysis. As the results of LASSO can be unreliable in the presence of collinear predictors, the correlations between all numerical predictors were checked prior to analysis, with the correlation matrix presented in SI Figure 2. If two variables were highly correlated (i.e., r ≥ 0.8), one of these variables was removed. Given the high correlation between total and LDL cholesterol (Pearson's r = 0.95), the former was removed from the list of predictors submitted to the LASSO model.

Failure to account for competing risk can inflate the associations between predictors and outcomes. A competing risk regression models the effect of predictors on the outcome while accounting for competing risk of death without dementia [26–28]. To obtain the coefficients of predictors, a competing risk regression was used with LASSO selected variables as predictors. Time at risk was derived as previously described (Follow up and censoring derivation). We used the crr command from the R package cmprsk. The resulting coefficients are used to compute predicted risk via the formula:

$$1 - S_0^{e^{(LP)}}$$

Where LP is the linear predictor and S_0 the baseline 14-year survival of an individual with all coefficients equal to 0 - i.e., being female, with the mean age of the training set (59.97 years), the mean years of education of the training set, (13.54 years) no diabetes, no depression, no stroke, not living alone, not materially deprived, and without parental history, hypertension, or hypercholesterolemia.

Model assumptions were checked by fitting a cox regression model with the selected predictors. The p-value for deviation from proportional hazards was significant for age and depression. However visual inspection of schoenfeld residuals showed minimal trend for both variables (SI Figure 3). For further inspection of depression, we plotted time vs. survival for those with/without depression. The survival curves for the two groups were parallel and did not cross, which indicates proportional hazards are satisfied. We also compared the form of the curves to those for the diabetes and family history variables, which did not have a significant p-value (SI Figure 4). In both cases, the curves showed similar form to depression, increasing confidence that assumptions were met. This led us to disregard the proportionality violations for these two predictors.

Evaluation of the UKBDRS

The performance of the UKBDRS was compared to the DRS, CAIDE, and ANU-ADRI in the UKB train, test and WHII datasets. All models were additionally compared to a baseline model consisting of chronological age only, to examine the added predictive value of additional factors. The model discrimination was evaluated using the area under the curve (AUC). To obtain an AUC, first a receiver operating curve (ROC) is created by plotting the sensitivity vs. specificity of a test. The points along the ROC curve correspond to sensitivity and specificity obtained with various decision thresholds. AUC is then computed as the area under the ROC. The AUC is interpreted as the probability that for a pair of individuals of whom one develops the outcome and the other either develops the outcome at a later time or not at all, the predicted risk is greater in an individual who develops dementia earlier [27]. In the UKB, the maximum follow up time was 14.8 years, and so we evaluate our score at a time horizon of 14 years. In the WHII, we evaluated AUC at 17 years as participants were younger at baseline and relatively few individuals had developed dementia at shorter time windows. By 17 years, 71 WHII participants had a dementia diagnosis. The longer follow-up in WHII allowed sufficient cases of dementia in the analyses

We used risk-calibration to assess the agreement between the observed proportion of dementia cases and predicted probabilities of developing dementia as calculated from the risk score. In a well calibrated model, it is expected that 25% of individuals with a predicted risk of 25% will develop the outcome. Conversely, in a poorly calibrated model, of those with a 25% predicted risk, 10% or 50% may actually develop the outcome. Participants were stratified into 10 risk groups based on their predicted probability of developing dementia. For each group, the mean predicted risk is then plotted against the observed risk of the group, calculated using the cumulative incidence function [29, 30]. A line of best fit of these 10 data points is then used to assess calibration. Perfect calibration is measured with a diagonal line (i.e. a slope of 0, intercept of 1), indicating that the proportion of individuals in each group who develop the outcome is in line with the average predicted risk of the group. Deviations from an intercept and slope of 0/1 indicate either over- or under-estimation of risk. As the WHII study did not have the minimum number of cases required to derive precise calibration intercepts and slopes (i.e., minimum n=100 dementia cases required) or calibration curves (minimum n=200 dementia cases required [31]), we only examined calibration in the UKB test set (SI Table 9, SI Figure 5).

Supplementary Information - Results

SI Figure 1: Participant flowchart for UK Biobank and Whitehall.

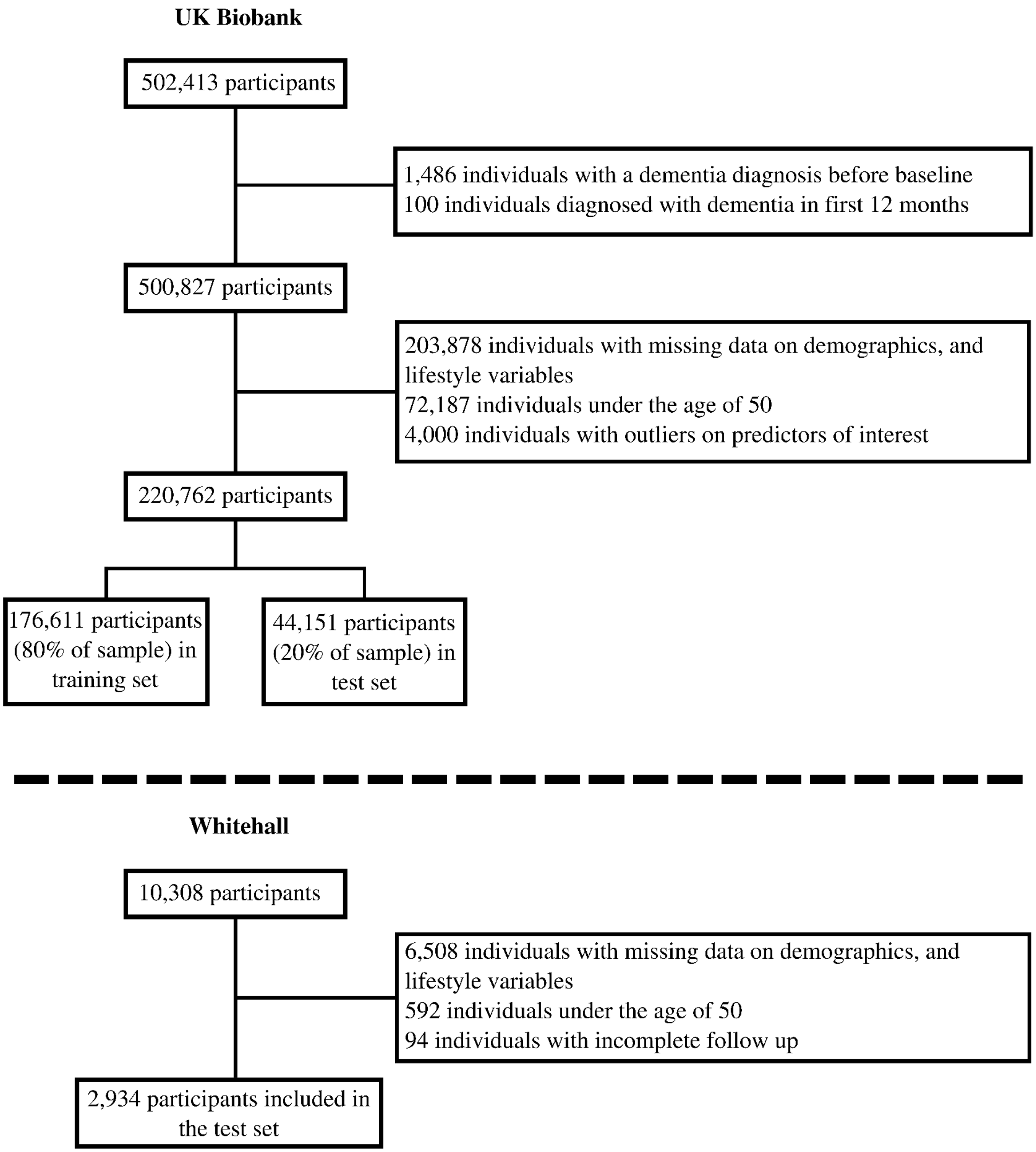

SI Table 1: Summary of information used for dementia ascertain across the two cohorts. ICD-9 codes were included to enhance our sensitivity to detecting historic cases of dementia prior to the adoption of ICD-10 codes for disease classification in the UK.

	UKB	WHII
ICD-9 all-cause dementia	290.2, 290.3, 290.4, 291.2, 294.1, 331.0, 331.1, 331.2, 331.5, 332	290.0, 290.1, 290.2, 290.3, 290.4, 331.0, 331.1, 331.2, 331.82, 331.9
ICD-10 all-cause dementia	A810, F00, F000, F001, F002, F009, F01, F010, F011, F012, F013, F018, F019, F02, F020, F021, F022, F023, F024, F028, F03, F051, F106, G30, G300, G301, G308, G309, G310, G311, G318, I673	F00, F01, F03, G30, G31
Self-report	Non-cancer Illness codes: ‘Parkinsons Disease’ (1262), ‘dementia/alzheimers/cognitive impairment; (1263)	Anyone who indicated that they have a longstanding illness of dementia
Medications	Compound names: Donepezil, Rivastigmine, Galantamine, Modafinil, Memantine	

SI Table 2: An overview of the candidate predictors considered for the UKB-DRS.

Variables	Description	UKB field
Demographic		
Age	Age was collected as baseline, in years.	21003
Education	Self-reported years of education was estimated based on an individual's highest educational qualification and the age at which they completed full-time education.	845, 6138
Sex	Females were coded as "0" and males were coded as "1".	31
Townsend Deprivation	Townsend Deprivation was calculated before participant enrolment, using national census output areas. Individuals were then assigned a score based on the location of their postcode within the output areas.	189
Biomedical/Medical		
BMI	Calculated from height and weight collected at baseline.	21001
Systolic BP	Two automated readings (a few moments apart) of systolic blood pressure were taken. The mean across these two measurements were used, unless one measurement was missing.	4080
History of Diabetes (Type I or Type II)	Diabetes was ascertained through self-report ("Has a doctor ever told you that you have diabetes?") and primary care data (ICD9: 250,2500,2501-2507,2509 and ICD10: E10, E100-E109, E11, E110-E119, E13,	2443, 42040

	E130-E139, E14, E140-E149).	
Total Cholesterol	Blood samples (non-fasting) were collected and assayed with a Beckman Coulter AU5800 analytical platform to determine total, HDL and LDL levels.	30690
HDL Cholesterol	As above.	30760
LDL Cholesterol	As above.	30780
Depression	Current or a history of depression was ascertained based on self-report (codes: 1286, 1291, 153) and ICD-9 (3004, 296, 2960-2966, 2968, 311) and ICD-10 codes (F320-F323, F328-F334, F338, F339).	20002, 42040
History of TBI	A history of traumatic brain injury (TBI) was determined through primary care data using ICD-9 (850-854, 800-804) and ICD-10 codes (S020-S029, S060-S071, S078-S079, S097-S099, T04, T06).	42040
History of Stroke/TIA	History of stroke and/or a transient ischemic stroke (TIA) was ascertained through self-report (**stroke**: 1583, 1081; **TIA**: 1082) and primary care data, using ICD-9 (**stroke**: 430, 431, 432, 436, 4320, 4321, 4329, 4330-4333, 4338-4341, 4349; **TIA**: 850-854, 800-804) and ICD-10 codes (**stroke**: I600-I619, I630-I649; **TIA**: G450-G454, G458, G459).	20002, 42040
History of Atrial Fibrillation	Atrial Fibrillation history was determined using self report (1471, 1483) and primary care variables (ICD-9: 42731,	20002, 42040

	42732; ICD-10: I480, I481, I482, I4891).	
Hypertension status	Hypertension status was determined based on self report (codes 1065,1072, 1073), ICD-10 (codes I10, I11, I12, I13, I15, I674), or self reported use of antihypertensive medication	20002, 42040
High cholesterol	Cholesterol status was determined based on self report (codes 1473), ICD-10 (codes E7780, E782, E784, E785), or self reported use of statins	20002, 42040
Parental History of Dementia	Participants were asked to list any illnesses that either their mother or father had.	20107, 20110
Hearing problems	Participants were asked whether they had difficulty with their hearing. Participants who indicated "Yes" or "I am completely deaf" were coded as "1" while those indicating "No" were coded as "0".	2247
Statins	Statin-use, hormone replacement therapy, anti-hypertensive medications, NSAIDs and aspirin use were each ascertained through *current* self-reported medications, based on the ATC codes reported in Supplementary Data 1 of Wu et al. (2019).	20003
Hormone Replacement Therapy		
Anti-hypertensive medications		
NSAIDs (excluding Aspirin)		
Aspirin		
Genetic		
APOE4	Genotype data was collected using the Affymetrix UK Biobank Axiom Array or UK BiLEVE Axiom array. APOE4 status was determined based on two single nucleotide	affy16020316, affy16020324

	polymorphisms: rs429358 and rs7412. An individual with one or more E4 allelles was coded as a carrier (="1").	
Lifestyle		
Smoking status	Participants reporting "ever" or "current" smokers were coded as "1" while those who did not smoke were coded as "0".	20116
Alcohol consumption	Alcohol intake was computed as weekly units per week.	1568, 1578, 1588, 1598, 5364, 1608, 4407, 4418, 4429, 4440, 4462, 4451, 20117, 20406
Physical Activity	IPAQ classifications of physical activity groups were used, with "moderate" to "high" activity groups coded as 1, and "low" coded as "0". For more details on these variables, please see Cassidy et al. (2016).	22032
Sleep duration (hours/ night)	Sleep duration was measured with the item "About how many hours sleep do you get in every 24 hours?"	1160
Sleeplessness	Participants were asked "Do you have trouble falling asleep at night or do you wake up in the middle of the night?" with the response options ranging from "Never/rarely" to "Usually".	1200
Frequency of family/ friend visits	This item asked individuals to report how often they visited or received visits from their family and friends. The response options ranged from: "No friends/family outside household" and "Never or almost never" to "Almost daily".	1031

Household occupancy	This item required respondents to indicate "Including yourself, how many people are living together in your household? (Include those who usually live in the house such as students living away from home during term, partners in the armed forces or professions such as pilots)". From this, participants were grouped into one of three categories - lives alone, lives with one other person, lives with multiple people.	709
Fish intake	Participants were asked to report their weekly oily (e.g., sardines, salmon, mackerel) and non-oily (e.g. cod, tinned tuna, haddock) fish intake.	1339, 1329

SI Figure 2: Heat map of correlations between continuous predictors of interest in the UKB training set. Given the high correlation between Total cholesterol and LDL, the former variable was removed from the list of predictors submitted to LASSO. **Abbreviations:-** BMI = Body Mass Index, LDL = Low-density Lipoprotein, HDL = High-density Lipopotein, SBP = Systolic Blood Pressure.

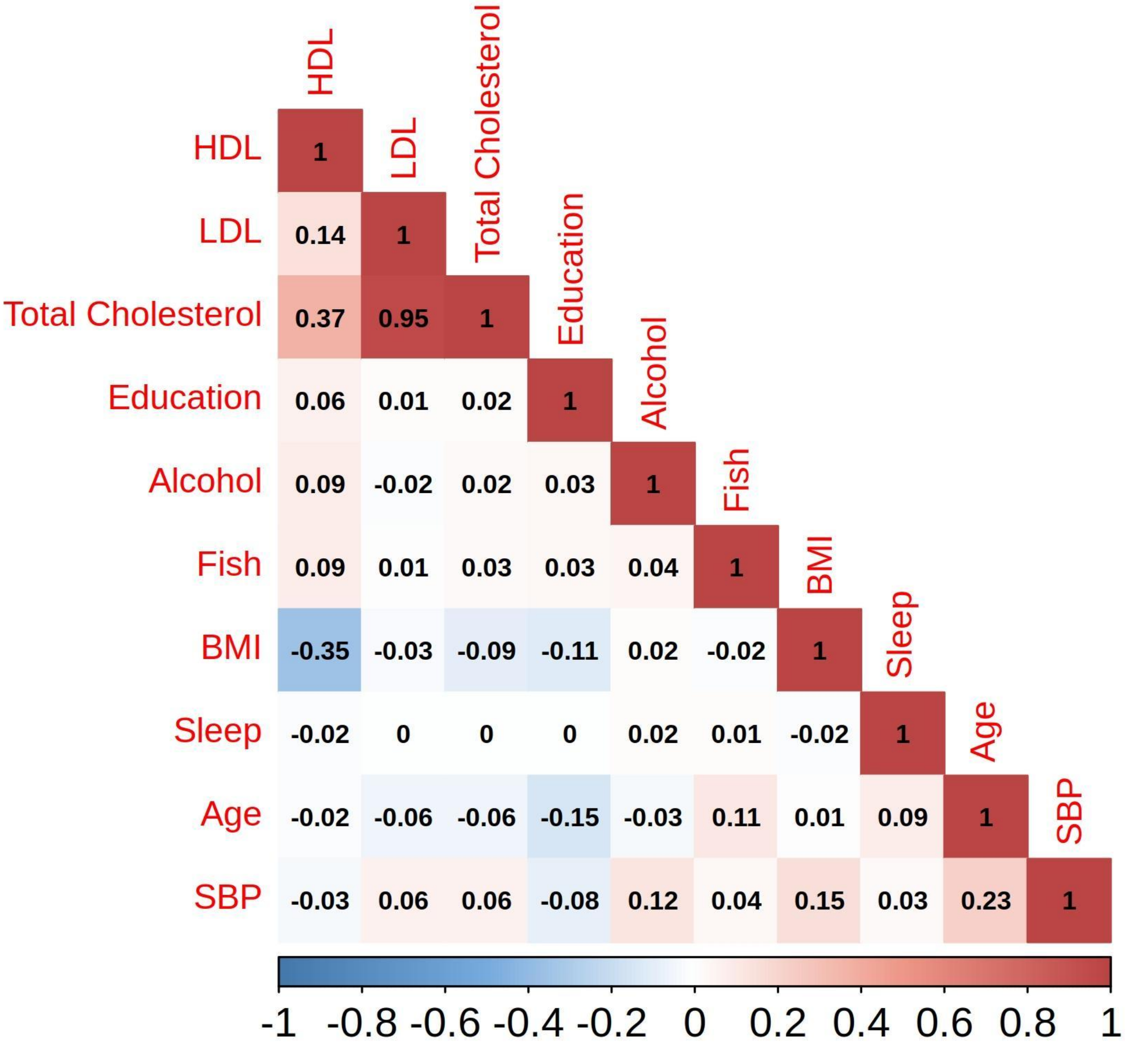

SI Figure 3: Schoenfeld Residuals: Plots of schoenfeld residuals vs time for each variable, extracted from a fitted cox model. Linear trends indicate deviation from proportional hazards. Marginal deviation observed for age and depression (top row), none for other variables such as education and hypertension (bottom row).

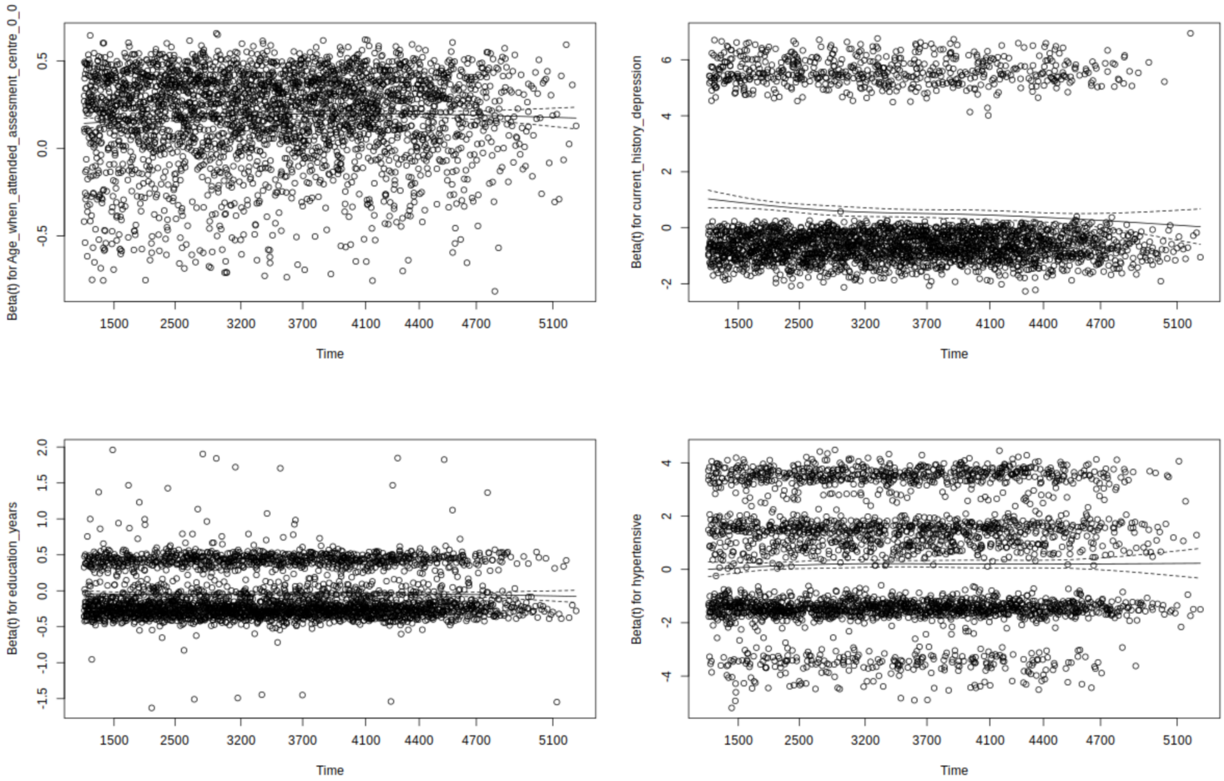

SI Figure 4: Survival curves for depression, diabetes, family history. To further explore potential non-proportional hazards, the survival curves of depression status (yes/no) was plotted, along with the log of survival time vs. log(-log(survival time)) (top row). Both show parallel lines and similar forms for survival vs. time for those with and without depression, indicating proportionality is met. The same plots for diabetes (middle row) and family history (bottom row) are shown for comparison.

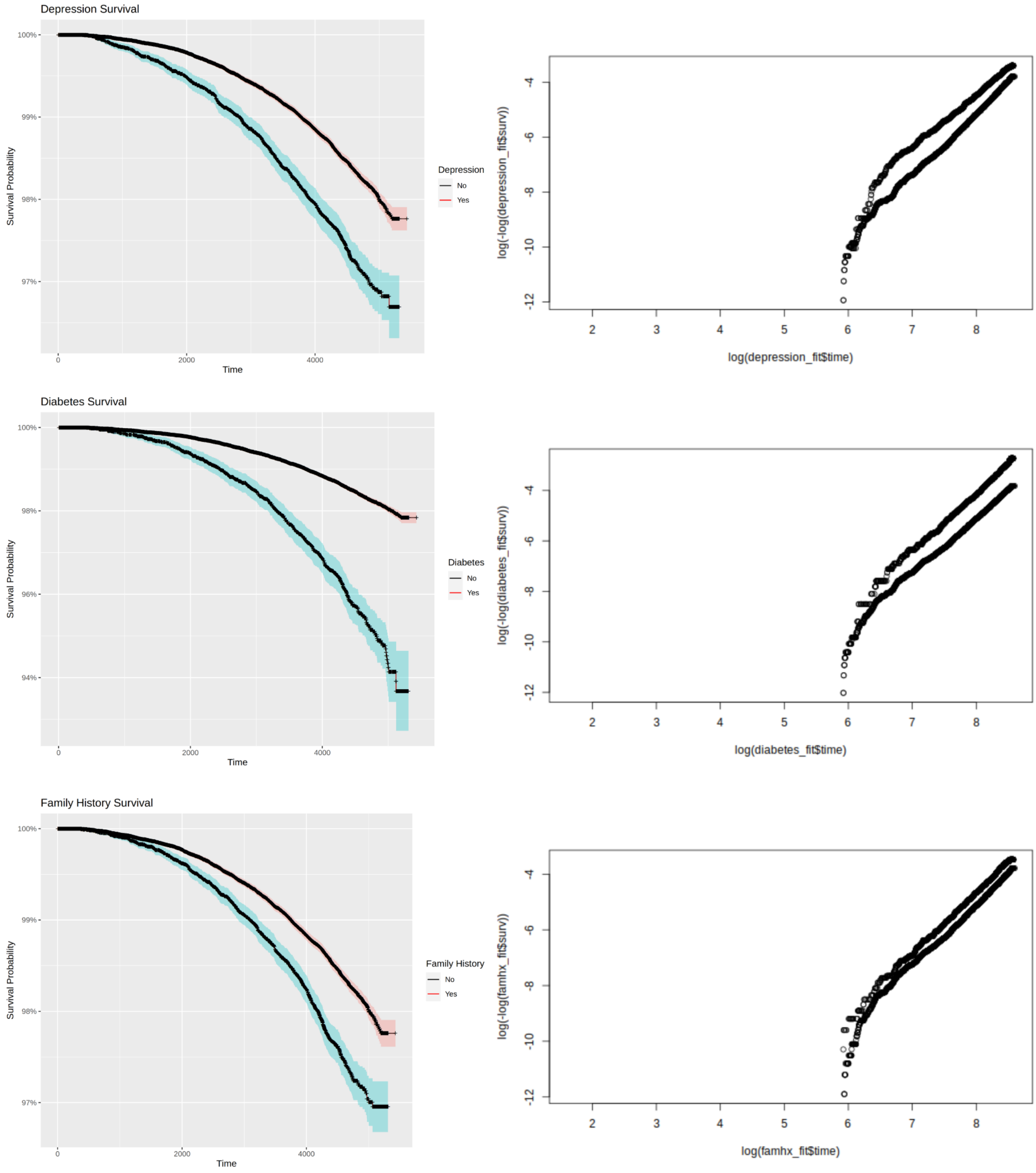

SI Table 3: Baseline characteristics of the UKB training set and UKB and WHII test sets. **Abbreviations**: APOE4 = Apolipoprotein E4, Body Mass Index = BMI, BP = Blood Pressure, HDL = High-density Lipoprotein, HRT = Hormone Replacement Therapy, IPAQ = International Physical Activity Questionnaire, IQR = Interquartile Range, LDL = Low-density Lipoprotein, N = Number, TIA = Transient Ischaemic Attack, TBI = Traumatic Brain Injury. Note: Continuous variables are reported as either Mean±SD or Median (IQR). Categorical variables are reported as N. (%). Percentage of APOE carriers is computed without considering those with unknown genetic status. A description of variables and how they were collected (as well as mapping to the relevant UKB field code) is available in SI Table 2.

	**UK Biobank**		**WHII**	
	**No dementia**	**Incident dementia**	**No dementia**	**Incident dementia**
**N**	**216,949**	**3,813**	**2,841**	**93**
**Demographic**				
Age at baseline, years	59.90 (±5.42)	64.47 (±4.18)	57 (IQR=10)	62 (IQR = 0)
Female (%)	111,578 (51.43%)	1,698 (44.53%)	802 (28.23%)	32 (34.41%)
Education, years	13.56 (±3.05)	12.72 (±3.10)	14.92 (±4.14)	14.25 (±4.19)
Townsend Deprivation				
Group 1: least deprived	43,758 (20.17%)	688 (18.04%)	1048 (36.89%)	30 (32.26%)
Group 2	43,710 (20.15%)	696 (18.25%)	1012 (35.62%)	32 (34.41%)
Group 3	43,498 (20.05%)	744 (19.51%)	621 (21.86%)	20 (21.51%)
Group 4	43,360 (19.99%)	751 (19.70%)	160 (5.63%)	11 (11.83%)
Group 5: most deprived	42,623 (19.65%)	934 (24.50%)		-
**Biomedical**				
BMI, kg/m^2	27.31 (±4.42)	27.49 (±4.62)	26.15 (±3.75)	26.80 (±4.36)
Systolic BP, mm Hg	140.01 (±18.41)	144.12 (±18.96)	124.74 (±16.57)	127.47 (±16.91)
Cholesterol				

Total, mmol/L	5.75 (±1.16)	5.53 (±1.29)	6.01 (±1.03)	6.07 (±1.12)
HDL, mmol/L	1.46 (±0.39)	1.43 (±0.41)	1.47 (±0.40)	1.46 (±0.43)
LDL, mmol/L	3.60 (±0.88)	3.44 (±0.96)	-	-
**Lifestyle**				
Sleep duration, hours/night	7.17 (±1.04)	7.25 (±1.18)	7 (IQR = 1)	7 (IQR = 1)
IPAQ group				
Low	40,241 (18.55%)	728 (19.09%)	655 (23.06%)	28 (30.11%)
Moderate-vigorous	176,708 (81.45%)	3,085 (80.91%)	2186 (76.94%)	65 (69.89%)
Alcohol intake, units/week	14.40 (±15.49)	13.20 (±15.91)	19.01 (±19.94)	14.86 (±19.15)
Current/previous smoker	101,968 (47.00%)	2,083 (54.63%)	237 (8.34%)	9 (9.68%)
Weekly fish intake	3.53 (±1.38)	3.66 (±1.45)	7.86 (±3.77)	7.43 (±4.10)
N. in household	2.25 (±1.20)	1.97 (±1.08)	2.63 (±1.25)	2 (±1.15)
N. of weekly leisure activities	1.05 (±0.87)	0.96 (±0.85)	1.48 (±0.99)	1.47 (±1.05)
Insomnia (sleeplessness)				
Never	50,351 (23.21%)	972 (25.49%)	-	-
Sometimes	103,994 (47.93%)	1,736 (45.53%)	-	-
Usually	62,604 (28.86%)	1,105 (28.98%)	-	-
**Genetic**				
APOE4 status (≥1 risk allele)	45,965 (29.8%)	1623 (56.8%)	551 (24.50%)	35 (53.03%)
**Medical**				
Depression	28,307 (13.05%)	729 (19.12%)	528 (18.59%)	23 (24.73%)
Diabetes	11,994 (5.53%)	505 (13.24%)	65 (2.29%)	6 (6.45%)

Stroke/ TIA	4,156 (1.92%)	231 (6.06%)	28 (0.99%)	1 (1.08%)
TBI	3,971 (1.83%)	107 (2.81%)	-	-
Atrial Fibrillation	2,839 (1.31%)	104 (2.73%)	-	-
Statins	42,195 (19.45%)	1,337 (35.06%)	-	-
NSAIDs	41,158 (18.97%)	711 (18.65%)	-	-
Aspirin	34,507 (15.91%)	1,097 (28.77%)	-	-
HRTs	8,957 (4.13%)	121 (3.17%)	-	-
Hypertensive	72,941 (33.62%)	1,889 (49.54%)	855 (30.10%)	34 (36.56%)
High Cholesterol	47,110 (21.71%)	1,445 (37.9%)	1070 (37.66%)	39 (41.94%)
Parental history of dementia	35,935 (16.56%)	893 (23.42%)	169 (5.95%)	11 (11.83%)
Time at risk	12.30 (±1.79)	8.87 (±2.96)	16.47 (±3.23)	13.08 (±3.66)

SI Table 4: Comparison of included and excluded participants. Note: Mean ± Standard Deviation, Median (IQR) or Frequency (%) are reported.

	**Included**	**Excluded**	**Test-statistic**	***p***
**UKB**				
Age	59.97 ± 5.43	60.21 ± 5.46	t = 13.31	2.08×10^{-40}
N. of females (%)	113,276 (51%)	95,444 (58%)	X^2= 1819.4	0
Education	13.54 ± 3.05	12.98 ± 3.07	t = -55.71	0
**WHII**				
Age	57 (IQR = 10)	57 (IQR = 10)	W = 4732034	8.88×10^{-3}
N. of females (%)	834 (28.43%)	1,128 (17.95%)	X^2 =20.19	7.00×10^{-6}
Education	14.89 ± 4.14	14.70 ± 4.49	t = -1.71	0.087

SI Table 5. The set of predictors selected in the LASSO model

Term	Estimate
Age	0.88
Diabetes	0.53
Depression	0.37
Stroke	0.61
Hypertensive	0.11
High Cholesterol	0.09
Townsend Deprivation (most deprived)	0.12
Education	-0.06
Sex (male)	0.03
Household Occupancy	0.01
Parental History	0.22

SI Table 6: Sensitivity Analyses. Discrimination of each model when predicting dementia within varying time frames. AUCs shown correspond to the UKB Test set.

	**Dementia within 1-5 years (n = 99)**	**Dementia within 5-10 years (n = 401)**
Age only	0.72 [0.67, 0.77]]	0.73 [0.71, 0.75]
UKBDRS	0.75 [0.71, 0.8]	0.76 [0.74, 0.78]
CAIDE	0.56 [0.51, 0.61]	0.58 [0.55, 0.6]
DRS	0.73 [0.69, 0.77]	0.73 [0.71, 0.75]
ANU-ADRI	0.54 [0.48, 0.59]	0.58 [0.55, 0.6]

SI Table 7: Discrimination accuracy for the supplementary stratification analysis. AUCs and 95% confidence intervals for subsets of the UK Biobank and WHII samples which were restricted to match the age range of the cohort in which the external score was originally developed and validated (listed in brackets beside external risk score names). The ANU ADRI was initially validated in three cohorts, two of which (MAP, CVHS) had overlapping age ranges with the UK Biobank sample. In bold are the UKBDRS (recommended model) and the respective reference risk score.

	**UKB Train**	**UKB Test**	**WHII**
**CAIDE (39-64 years)**	N = 133,965	N = 33,476	N = 2,643
UKBDRS	0.77 [0.77, 0.77]	0.77 [0.74, 0.8]	0.77 [0.72, 0.83]
CAIDE	0.61 [0.61, 0.61]	0.62 [0.59, 0.66]	0.71 [0.66, 0.77]
**DRS (60-79 years)**	N = 98,697	N = 24,794	N = 1,081
UKB-DRS	0.69 [0.69, 0.69]	0.71 [0.69, 0.74]	0.59 [0.52, 0.66]
DRS	0.65 [0.65, 0.65]	0.67 [0.64, 0.69]	0.53 [0.45, 0.6]
**ANU ADRI (MAP) (53-100 years)**	N =147,277	N = 36,912	N = 1,828
UKB-DRS	0.75 [0.75, 0.75]	0.77 [0.75, 0.79]	0.69 [0.64, 0.74]
ANU_ADRI	0.56 [0.56, 0.56]	0.57 [0.54, 0.6]	0.55 [0.48, 0.61]
**ANU ADRI (CVHS) (60-100 years)**	N = 98,697	N = 24,794	N = 1,081
UKB-DRS	0.69 [0.69, 0.7]	0.71 [0.69, 0.74]	0.59 [0.52, 0.66]
ANU_ADRI	0.58 [0.58, 0.58]	0.59 [0.56, 0.62]	0.59 [0.52, 0.66]

SI Table 8: Risk score cut-offs at 80%, 85%, 90% and 95% sensitivity and specificity. Note: the predicted probabilities of risk have been converted to percentages to aid in interpretation. Abbreviations:- NPV = Negative predictive value; PPV = Positive predictive value.

	**Cut-off (%)**	**Specificity**	**Sensitivity**	**NPV**	**PPV**
**UKBDRS**					
Sensitivity:					
80%	1.843	0.642	-	0.995	0.038
85%	1.451	0.569	-	0.995	0.034
90%	1.140	0.499	-	0.997	0.031
95%	0.671	0.345	-	0.997	0.025
Specificity:					
80%	3.201	-	0.585	0.991	0.049
85%	3.848	-	0.493	0.99	0.055
90%	4.804	-	0.37	0.988	0.061
95%	6.373	-	0.236	0.986	0.076
**UKBDRS-APOE**					
Sensitivity:					
80%	1.862	0.676	-	0.995	0.042
85%	1.458	0.607	-	0.996	0.037
90%	1.00	0.504	-	0.997	0.031
95%	0.567	0.343	-	0.997	0.025
Specificity:					
80%	3.038	-	0.661	0.993	0.055
85%	3.746	-	0.591	0.992	0.065
90%	5.052	-	0.477	0.99	0.077
95%	7.563	-	0.321	0.988	0.101

SI Table 9: Calibration intercept and slope. The mean predicted vs. observed risk is plotted within 10 risk stratifications, and linear regression is used to fit the intercept and slope of the line. Deviations from intercept/slope of 0/1 indicate imperfect calibration.

**Model**	Intercept [95% CI]	Slope [95% CI
UKBDRS	-0.001 [-0.004, 0.002]	1.025 [0.93, 1.12]
UKBDRS-APOE	0.001 [-0.003, 0.006]	0.969 [0.851, 1.087]

SI Figure 5: Calibration curves for each of the risk scores assessed in the UKB Test set.

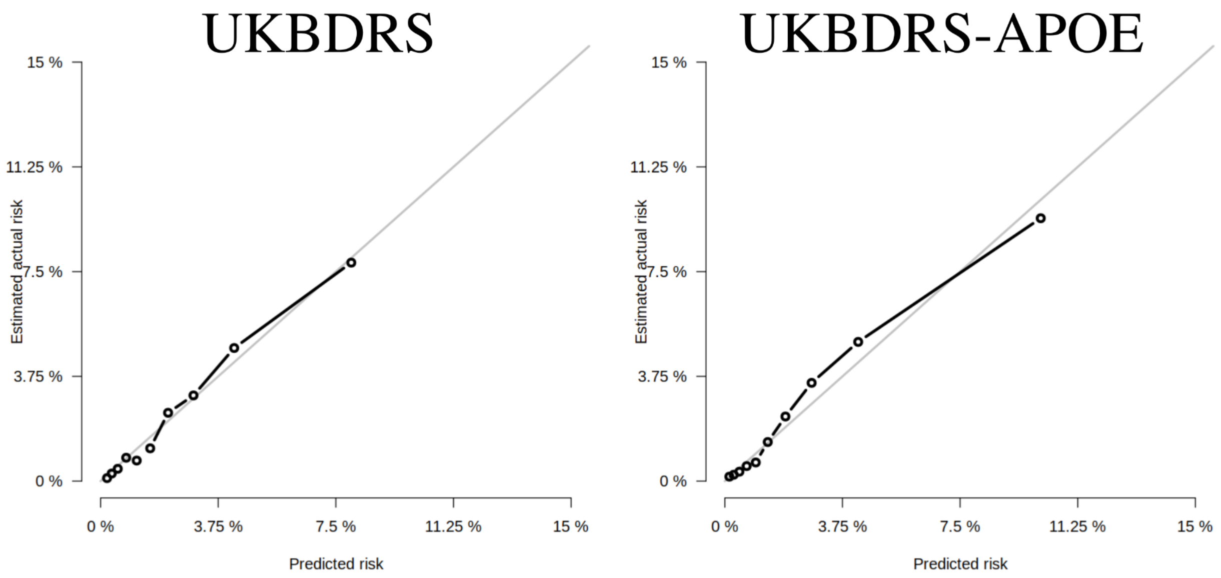

References

1. Sudlow C, Gallacher J, Allen N, et al (2015) UK biobank: an open access resource for identifying the causes of a wide range of complex diseases of middle and old age. PLoS Med 12:e1001779

2. Marmot M, Brunner E (2005) Cohort Profile: the Whitehall II study. Int J Epidemiol 34:251–256

3. Sabia S, Fayosse A, Dumurgier J, Schnitzler A, Empana J-P, Ebmeier KP, Dugravot A, Kivimäki M, Singh-Manoux A (2019) Association of ideal cardiovascular health at age 50 with incidence of dementia: 25 year follow-up of Whitehall II cohort study. BMJ 366:l4414

4. Petermann-Rocha F, Lyall DM, Gray SR, Esteban-Cornejo I, Quinn TJ, Ho FK, Pell JP, Celis-Morales C (2020) Associations between physical frailty and dementia incidence: a prospective study from UK Biobank. The Lancet Healthy Longevity 1:e58–e68

5. Gong J, Harris K, Peters SAE, Woodward M (2021) Sex differences in the association between major cardiovascular risk factors in midlife and dementia: a cohort study using data from the UK Biobank. BMC Med 19:110

6. Zhang H, Greenwood DC, Risch HA, Bunce D, Hardie LJ, Cade JE (2021) Meat consumption and risk of incident dementia: cohort study of 493,888 UK Biobank participants. Am J Clin Nutr 114:175–184

7. Lourida I, Hannon E, Littlejohns TJ, Langa KM, Hyppönen E, Kuzma E, Llewellyn DJ (2019) Association of Lifestyle and Genetic Risk With Incidence of Dementia. JAMA 322:430–437

8. Tai XY, Veldsman M, Lyall DM, Littlejohns TJ, Langa KM, Husain M, Ranson J, Llewellyn DJ (2022) Cardiometabolic multimorbidity, genetic risk, and dementia: a prospective cohort study. Lancet Healthy Longev 3:e428–e436

9. Shang X, Hill E, Zhu Z, Liu J, Ge BZ, Wang W, He M (2021) The Association of Age at Diagnosis of Hypertension With Brain Structure and Incident Dementia in the UK Biobank. Hypertension 78:1463–1474

10. Malik R, Georgakis MK, Neitzel J, Rannikmäe K, Ewers M, Seshadri S, Sudlow CLM, Dichgans M (2021) Midlife vascular risk factors and risk of incident dementia: Longitudinal cohort and Mendelian randomization analyses in the UK Biobank. Alzheimers Dement 17:1422–1431

11. Sabia S, Fayosse A, Dumurgier J, van Hees VT, Paquet C, Sommerlad A, Kivimäki M, Dugravot A, Singh-Manoux A (2021) Association of sleep duration in middle and old age with incidence of dementia. Nat Commun 12:2289

12. Abell JG, Kivimäki M, Dugravot A, Tabak AG, Fayosse A, Shipley M, Sabia S, Singh-Manoux A (2018) Association between systolic blood pressure and dementia in the Whitehall II cohort study: role of age, duration, and threshold used to define hypertension. Eur Heart J 39:3119–3125

13. Sabia S, Dugravot A, Dartigues J-F, Abell J, Elbaz A, Kivimäki M, Singh-Manoux A (2017)

Physical activity, cognitive decline, and risk of dementia: 28 year follow-up of Whitehall II cohort study. BMJ 357:j2709

14. Livingston G, Huntley J, Sommerlad A, et al (2020) Dementia prevention, intervention, and care: 2020 report of the Lancet Commission. Lancet
15. Craig CL, Marshall AL, Sjöström M, et al (2003) International physical activity questionnaire: 12-country reliability and validity. Med Sci Sports Exerc 35:1381–1395
16. Clarke T-K, Adams MJ, Davies G, et al (2017) Genome-wide association study of alcohol consumption and genetic overlap with other health-related traits in UK Biobank (N=112 117). Mol Psychiatry 22:1376–1384
17. Topiwala A, Wang C, Ebmeier KP, et al (2022) Associations between moderate alcohol consumption, brain iron, and cognition in UK Biobank participants: Observational and mendelian randomization analyses. PLoS Med 19:e1004039
18. Anstey KJ, Cherbuin N, Herath PM (2013) Development of a New Method for Assessing Global Risk of Alzheimer's Disease for Use in Population Health Approaches to Prevention. Prevention Science 14:411–421
19. Licher S, Yilmaz P, Leening MJG, Wolters FJ, Vernooij MW, Stephan BCM, Ikram MK, Ikram MA (2018) External validation of four dementia prediction models for use in the general community-dwelling population: a comparative analysis from the Rotterdam Study. Eur J Epidemiol 33:645–655
20. Walters K, Hardoon S, Petersen I, Iliffe S, Omar RZ, Nazareth I, Rait G (2016) Predicting dementia risk in primary care: development and validation of the Dementia Risk Score using routinely collected data. BMC Med 14:6
21. Kivipelto M, Ngandu T, Laatikainen T, Winblad B, Soininen H, Tuomilehto J (2006) Risk score for the prediction of dementia risk in 20 years among middle aged people: a longitudinal, population-based study. Lancet Neurol 5:735–741
22. Licher S, Leening MJG, Yilmaz P, et al (2019) Development and Validation of a Dementia Risk Prediction Model in the General Population: An Analysis of Three Longitudinal Studies. Am J Psychiatry 176:543–551
23. Nori VS, Hane CA, Martin DC, Kravetz AD, Sanghavi DM (2019) Identifying incident dementia by applying machine learning to a very large administrative claims dataset. PLoS One 14:e0203246
24. Pavlou M, Ambler G, Seaman SR, Guttmann O, Elliott P, King M, Omar RZ (2015) How to develop a more accurate risk prediction model when there are few events. BMJ 351:h3868
25. Tibshirani R (1996) Regression shrinkage and selection via the lasso. J R Stat Soc 58:267–288
26. Fine JP, Gray RJ (1999) A Proportional Hazards Model for the Subdistribution of a Competing Risk. J Am Stat Assoc 94:496–509
27. Blanche P, Dartigues J-F, Jacqmin-Gadda H (2013) Estimating and comparing time-dependent areas under receiver operating characteristic curves for censored event

times with competing risks. Stat Med 32:5381–5397

28. Ramspek CL, Teece L, Snell KIE, Evans M, Riley RD, van Smeden M, van Geloven N, van Diepen M (2022) Lessons learnt when accounting for competing events in the external validation of time-to-event prognostic models. Int J Epidemiol 51:615–625

29. Austin PC, Putter H, Lee DS, Steyerberg EW (2022) Estimation of the Absolute Risk of Cardiovascular Disease and Other Events: Issues With the Use of Multiple Fine-Gray Subdistribution Hazard Models. Circ Cardiovasc Qual Outcomes 15:e008368

30. Wolbers M, Koller MT, Witteman JCM, Steyerberg EW (2009) Prognostic models with competing risks: methods and application to coronary risk prediction. Epidemiology 20:555–561

31. Van Calster B, Nieboer D, Vergouwe Y, De Cock B, Pencina MJ, Steyerberg EW (2016) A calibration hierarchy for risk models was defined: from utopia to empirical data. J Clin Epidemiol 74:167–176
